# The Potential Mechanisms Behind Adverse Effect of Coronavirus Disease-19 on Heart and Liver Damage: A Review

**DOI:** 10.4314/ejhs.v34i1.10

**Published:** 2024-01

**Authors:** Tolessa Muleta Daba, Mulatu Mokonon, Elsa Niguse, Meron Getahun

**Affiliations:** 1 Deparment of Biochemistry, Molecular Biology and Genetics, School of Medicine and Pharmacy, College of Medicine and Health Sciences, University of Rwanda, Huye Campus, Rwanda; 2 Institute of Pharmaceutical Science, Adama Science and Technology University, Adama, Ethiopia; 3 Department of Biology, School of Applied Natural Sciences, Adama Science and Technology University, Adama, Ethiopia

**Keywords:** Acute Cardiac Injury, Angiotensin-converting enzyme, COVID-19, Multiple organ damage

## Abstract

**Background:**

Coronaviruses (CoVs) belong to the RNA viruses family. The viruses in this family are known to cause mild respiratory disease in humans. The origin of the novel SARS-COV2 virus that caused the coronavirus-19 disease (COVID-19) is the Wuhan city in China from where it disseminated to cause a global pandemic. Although lungs are the predominant target organ for Coronavirus Disease-19 (COVID-19), since its outbreak, the disease is known to affect heart, blood vessels, kidney, intestine, liver and brain. This review aimed to summarize the catastrophic impacts of Coronavirus disease-19 on heart and liver along with its mechanisms of pathogenesis.

**Methods:**

The information used in this review was obtained from relevant articles published on PubMed, Google Scholar, Google, WHO website, CDC and other sources. Key searching statements and phrases related to COVID-19 were used to retrieve information. Original research articles, review papers, research letters and case reports were used as a source of information.

**Results:**

Besides causing severe lung injury, COVID-19 has also been reported to affect and cause dysfunction of many other organs. COVID-19 infection can affect people by downregulating membrane-bound active angiotensin-converting enzyme (ACE). People who have deficient ACE2 expression are more vulnerable to COVID-19 infection. The patients' pre-existing co-morbidities are major risk factors that predispose individuals to severe COVID-19.

**Conclusion:**

The disease severity and its broad spectrum phenotype is a result of combined direct and indirect pathogenic factors. Therefore, protocols that harmonize many therapeutic preferences should be the best alternatives to de-escalate the disease and obviate deaths caused as a result of multiple organ damage and dysfunction induced by the disease.

## Introduction

*Coronaviruses (CoVs)* belong to RNA viruses family. The viruses in this family are known to cause mild human respiratory disease. In the last two decades, the virus was reported to cause disease outbreaks in East Asia and the Middle East. The severe acute respiratory syndrome (SARS) and Middle East Respiratory Syndrome (MERS) are among the two significant coronavirus diseases that occurred in 2002 and 2012, respectively (1,2). In the late 2019, the outbreak of a novel coronavirus disease named severe acute respiratory syndrome coronavirus 2 (SARS-CoV-2) was reported (3). The diseases created a global health crisis that resulted in a pandemic in many countries across the world. The disease was named (COVID-19) which is derived from coronavirus disease 2019 (4,5).

Initially, the virus was reported in Wuhan city in China from where it was disseminated worldwide. The virus shares genetic similarity with SARS coronavirus and bat SARS-like coronaviruses which are known to have positivesense single-stranded RNA (6,7). It is grouped under β family coronavirus. The virus particle consists of four structural proteins which include S (spike), E (envelope), M (membrane), and N (nucleocapsid), and has a diameter of 50-200 nm (8). The viral envelope is made up of S, E, and M proteins while N protein creates the RNA genome of the virus (9).

Bat and Malayan pangolin are reported to be the potential natural and intermediate hosts of SARS-CoV-2 respectively (10). The virus genetic analysis suggests that the bat is the most likely origin of SARS-CoV-2 (11). However, vague evidence suggests the presence of an intermediary animal host in the origins of the virus (12).

The respiratory system is the primary target of the virus where it is known to cause from mild flulike illness such as a cough and fever to more severe conditions such as breathing difficulty (13). Available data indicate that COVID-19 pathogenesis, severity and mortality is condition dependent of which age and other predisposing illnesses are known to complicate the disease. High mortality was reported in immunocompromised people and people greater than 60 years (14). In addition to acute respiratory distress syndrome and respiratory organ failure, COVID-19 is also known to cause systemic inflammation and myocarditis that can progress to acute cardiac injury followed by heart failure and other multiple organ damage and dysfunction in the patients (15). The pathogenesis of the disease varies from asymptomatic mild illness such as fever, fatigue, dry cough, and headache to severe complications such as respiratory system failure (5). The high rates of global mortality attributed to COVID-19 are caused by its effects causing multiple organ damage which occurs most frequently in people with other heath complications such as diabetes mellitus, hypertension, cardiovascular disease and advanced age (16). Even though the target organ for SARSCoV-2 is believed to be the lungs, currently other organs such as the heart, liver, kidney, stomach, and intestine are also reported to be affected by the virus (17).

Understanding the detailed pathogenesis mechanisms of the virus and its virulence is imperative and helps contribute to the development of effective therapeutic strategies. This review aimed at summarizing the available published information on catastrophic effect of COVID-19 on other organs, especially the heart and the liver along with mechanism of pathogenesis.

Information used in this review was obtained from relevant articles published on PubMed, NCBI, Google Scholar, Google, WHO website, CDC websites and others. Key searching statements and phrases such as mechanism of Covid-19 pathogenesis, genome structure, the adverse damage of Covid-19 on multiple organ, SARSCoV-2 and heart diseases, SARSCoV-2 and liver diseases, mechanism of Covid-19 disease progression to and liver, and others were used. Original research articles, systematic review papers and meta-analyses, research letters, and case reports closely related to the current review were considered.

### Sarscov-2 Pathogenesis and The Mechanisms Underlying Its Adverse Effects on Organ Damage

**The history of SARSCoV-2 Outbreak**: The new strain novel Coronavirus (CoV) was identified in Wuhan city in China and was named Wuhan virus to indicate the city where the initial outbreak occurred. The name was later changed to 2019 Novel Coronavirus or 2019-nCoV (18). Meanwhile, the disease got another name, Coronavirus disease 2019 (COVID-19) by combining ‘CO’ that stands for Corona, ‘VI’ for Virus, and ‘D’ for Disease (19). The naming is related to its outer surface crown-like spikes and hence named Coronavirus (7).

Like other members of the corona virus family, SARSCoV-2 is also known to cause severe acute respiratory syndrome (SARS) and also certainly causes other common colds (20). Following the first outbreak, the virus swiftly spread throughout China causing more than 81,552 cases (21). The virus was then immediately spread worldwide compelling the World Health Organization (WHO) to declare a public health emergency in late January 2020 and proclaim COVID-19 as a pandemic in March 2020 to curtail the geographical spread of the disease(19,21).

Coronaviruses are very small viruses that have a diameter of 65–125 nm. The virus nucleic acid is a single stranded RNA that ranges from 26 to 32kbs in length (7). The virus's family consists of four subgroups, alpha, beta, gamma and delta Coronavirus (22). Coronavirus family members are reported to cause different respiratory diseases, of which severe acute respiratory syndrome coronavirus (SARS-CoV), H5N1 influenza A, H1N1 2009 and middle east respiratory syndrome Coronavirus (MERS-CoV) are the commonest (23). Before the outbreak of severe acute respiratory syndrome (SARS) that was caused by SARS-CoV in Guangdong, China in 2002, it was thought that coronaviruses only infected animals (24). A decade after the SARS-CoV outbreak, another endemic coronavirus named middle east respiratory syndrome coronavirus (MERS-CoV) spread in middle eastern countries in 2012 (25).

Seven years later, another deadly novel coronavirus outbreak occurred again in Wuhan city in China towards the end of 2019. Reports showed that the Hunan seafood market in Wuhan city was the initial source of the virus (26). Further studies revealed that the virus is transmitted from animals to humans by consumption of infected animal carcasses and close contact with an infected person (27). In less than fifty days of outbreak, the virus had infected seventy thousand individuals causing thousands of deaths (7).

In late 2020 and early 2021, the disease transmission increased following emergence of a wide range of SAR-CoV-2 variants (28). The initial Wuhan strain was replaced with these rapidly transmissible variants that initiated successive waves of infections in many countries (29). In late 2020, the alpha variant was first emerged in Kent, UK and widely disseminated causing a second wave of infection in early 2021 (30). Following this second wave, another delta variant was discovered and was responsible for the third wave of infection that emerged and was first seen in India (31).

The fourth wave came following the emergency of the new Omicron variant in South Africa (32). This variant caused a resurgence of the COVID-19 incidence and was associated with a large number of mutations, including many in the spike protein which were associated with a higher rate of reinfection (29). Soon, the incidence of Omicron was increased in many countries all over the world (33).

**Figure 1:**
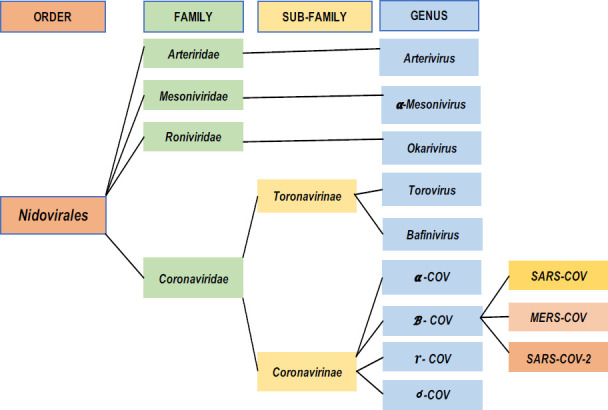
Types of coronaviruses (Modified from Zhou et al., 2021 (27). SARS-COV-2 with its genus members SARS-COV and MERS-COV are categorized under β - COV genus of Coronavirinae sub-family and Coronaviridae family together which all are under Nidovirales)

**Types of Coronavirus Diseases**: Coronavirus is among *Corona viridae* family of order *Nidovirales* that is composed of four genera which includes α-, β-, γ-, and δ Coronaviruses (Figure 1) (27). The α and β Coronaviruses are known to infect mammals, while γ Coronaviruses are the main causative agents of avian Coronavirus, whereas δ Coronaviruses are known to infect both mammals and aves (25,34). Until recently, the β coronavirus genus was known to cause different SARS-CoV's diseases such as mouse hepatitis coronavirus (MHV), MERS-CoV, bovine coronavirus (BCoV), bat coronavirus HKU4, and human coronavirus OC43, and SARS-CoV-2 (35). All diseases are transmitted from animals to humans when there is a close contact between humans and contaminated animals (36). It is in such a manner that SARS-CoV-2 swiftly spread in the population and exponentially projected the infected cases (6).

**Figure 2:**
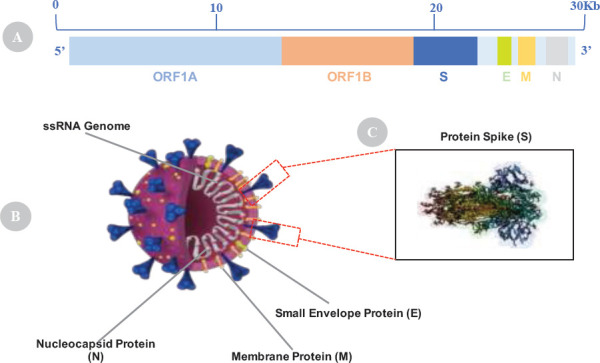
COVID-19 Genome Structure. Modified from Kumar et al.,2019 (39).Full-length ssRNACOVID-19. (B) COVID-19 viral particle which consists of ssRNA genome, Spike Protein (S), Envelope Protein (E), Membrane Protein (M) and Nucleocapsid Protein (N). (C) COVID-19 spike protein open conformation

**Genetic make-up of Corona Viruses**: Coronaviruses have crown-shaped spikes in their outer surface from which the virus gets its name (Figure 2) (7). The virus is an enveloped virus with 29.9 kb-long genome and positive-sense single-stranded(37). Of the whole virus genome, about 70% can form an open reading frame (ORF1a and 1b) that encodes and forms non-structural proteins (Figure 2A) (38). In addition, the viral genome encodes four main structural proteins which include Spike (S), Membrane (M), Envelope (E) glycoproteins, and Nucleocapsid (N) protein (Figures 2B and C) (38). Another fifth structural protein called Hemagglutinin Esterase (HE) is also coded in some beta coronaviruses.

About 82% sequence of the COVID-19 genome has similarity with SARS-CoV and MERS-CoV in general and 79% with the human coronaviruses SARS-CoV and 50% with MERS-CoV in particular (6). Comparative genome analysis revealed that the highest genetic resemblance is shared with bat RATG13 and Malayan pangolin coronavirus with approximate percentage similarity of 96 and 91% respectively (6).

**Figure 3:**
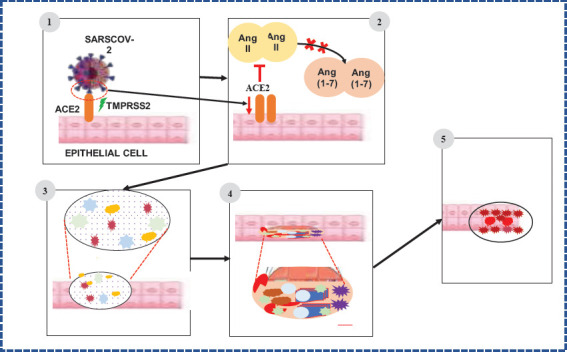
Pathogenesis of COVID-19- Modified from Naqvi et al.,2020 and Murgolo et al. 2021 (6,46). T-shape red arrow: inhibition of angiotensin-converting enzyme 2 cleavage to Ang (1-7); downward red arrow: down regulation of gene expression. For COVID-19 pathogenesis and progression five major pathological mechanisms involved. These includes: (1) Spike protein interaction with angiotensin-converting enzyme 2 (ACE2); (2) Downregulation of ACE2 expression and dysregulation of the reninangiotensin-aldosterone system caused by inhibited cleavage of angiotensin II (Ang II) to Ang (1-7); (3) overproduction of pro-inflammatory cytokines and chemokines that results in exorbitant acute inflammatory responses; (4) Intravascular thrombus formation caused by SARS-CoV-2 cytotoxic effects; and (5) Organ injury characterized by interstitial thickening, fibroblast proliferation, and fibrosis

**Mechanisms of Coronavirus Pathogenesis**: After infecting an individual, the virus attaches itself either to the surface of oral cavity epithelial membrane or mucosal membranes of the conjunctiva from where it enters the target organ via angiotensin-converting enzyme 2 (ACE2) (40). After entering into the epithelial cells of lung alveolar, the virus spike protein (S) interacts with ACE2 thus facilitating the binding of the virus to lung cells (Figure 3) (40). According to Trougakos and his co-authors, lung alveolar epithelial cells are the primary target of the virus since ACE2 is highly expressed (40). In the cell cytosol, the virus stimulates proteolytic cleavage of its S protein by using cellular enzyme, furin and the fusion of the virus and cellular membranes takes place (41).The virus S protein cleavage helps viral entry into the lung cells whereby this activated protein is primed by cellular transmembrane serine protease 2 (TMPRSS2) and attaches to ACE2 receptors that assist the entrance of the virus into the lung cell (42). The endosomal/lysosomal cysteine proteases cathepsin B and L (CTSB, CTSL) can also assist the entry of the virus into the cell (42,43). The S protein and cellular membrane fusion cause the release of the viral RNA genome into the cell cytoplasm.

The cell-surface ectopeptidases, such as dipeptidyl peptidase 4 (DPP4), aminopeptidase N, and angiotensin-converting enzyme 2 (ACE2) are also the most common cell-surface proteins onto which human coronaviruses commonly attach to enter the host cell (44). ACE2 is the key cell entry receptor for both SARS-CoV and SARS-CoV-2 (35,42). ACE2 presence in the host cell and individual variation in its expression can affect susceptibility to SARS-CoV-2 infection and organ injury consequent to the virus infection (45).

**Figure 4:**
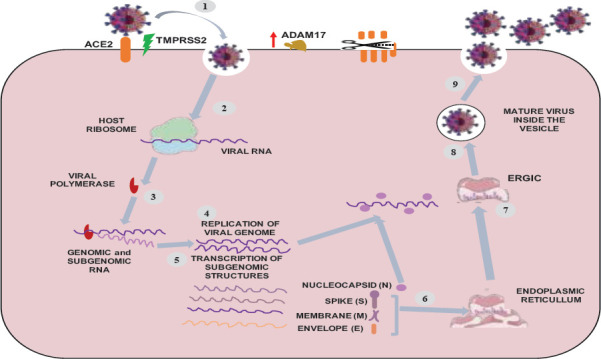
Mechanisms of SARS-CoV-2 infection and replication- Modified from Hoffman et al., 2020 and Jackson et al.,2022 (42,49). Upward red arrow: Upregulation of gene expression. (1) TMPRSS2 primes spike protein and facilitate its interaction with the ACE2, (2) Viral RNA-dependent RNA polymerase synthesis, (4) Replication of the SARS-CoV-2 genome and (5) sub genomic structures transcription (6) Endoplasmic reticulum assisted translation of sub-genomic transcripts to viral structural proteins. (7) Exporting of viral structures to the endoplasmic-reticulum-Golgi intermediated compartment (ERGIC). (8) New viral particles assembly (9) Exporting of mature virions to the extracellular compartment via exocytosis

After the entrance of the virus into the lung alveolar epithelial cells, replication of viral genome, viral RNA synthesis, viral RNA translation, viral replicase assembly, and assembly of newly synthesized virions in the endoplasmic reticulum-golgi intermediate compartment occur respectively. The assembled virions are then transported to the cell surface inside vesicles and released from the host cell by exocytosis (Figure 4) (2,47).

The mechanisms of SARS-CoV-2 interaction with the host is not well known. However, five major pathological mechanisms are known to be involved in COVID-19 development and progression (48). These include: (1) Cytotoxicity of ACE2-expressing cells initiated by virus; (2) Virus-mediated ACE2 downregulation that leads to RAAS dysregulation; (3) Abnormal immune responses; (4) Endothelial cell injury and thrombo-inflammation; and (5) Tissue fibrosis (48).

**Figure 5:**
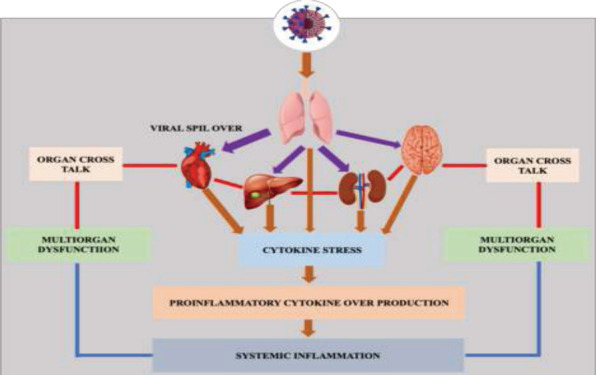
Mechanisms of Multiple organ injury and interferences in COVID-19 patients. Brown arrow indicates viral/ disease progression, purple arrow indicates viral spill over, red line indicate organ cross talk. The above figure shows multiple organ injury caused as result of COVID-19 diseases complication. Multiple organ injury complication is caused either as a result of direct viral spillover to the other organ or because of response to the disease. The two cases either separately or by collateral damage cause multiple organ deterioration. After the respiratory tract infection, SARS-CoV-2 virus replicate and viral spillover occur to the neighboring organs which directly bind to the organ via available receptor and start pathogenesis. In other way, the immune system response as result of respiratory tract infection cytokine stress which initiate the overproduction pro-inflammatory molecules and finally results in systemic infection of other organ. The cross talk between organs can also contribute for multiple organ damage following COVID-19 infection

After the virus replicates in the lung epithelial cell, ACE2 can be downregulated and prevents the split of angiotensin II into angiotensin (1–7) (Figure 5). According to Wang et al (2020), the virus is also known to invade the host cells via CD 147 receptors route (50). However, it is not clear whether other mediators are involved in the virus invading process of the host cells (51). The level of inflammatory cytokines generated indicate the severity of COVID-19 (52). For example, lymphopaenia is observed in patients with severe COVID-19. Likewise, significant lymphocytic T Cells (CD4+ and CD8+) and natural killer (NK) cells level reduction was observed in severe COVID-19 patients (53).

Both innate and adaptive immune systems are targeted during pathogenesis of SARS-CoV-2 (25,54). During viral infection, T cells and secondary messengers like cytokines play important roles in the progression of the disease (55). Likewise, an evolutionarily conserved innate pattern recognition receptors (PRRs) and humoral antibodies are also known to be involved and regulate the immune response of SARS-CoV-2 and the course of infection (6).

Hoffmann et al. (2020) reported that, currently, furin protease is also known to be involved in the pathogenesis of SARS-CoV-2 (42). This is achieved by the binding of cellular receptor neuropilin-1 with furin-cleaved substrates that pave a way into the central nervous system to effectuate SARS-CoV-2 infectivity. SARS-CoV-2 may also infect cerebral nervous system via receptor CD147 which is highly expressed in the brain (40). The viruses can be carried via the bloodstream and infect tissues with ACE2-expressing cells (5,17).

**Adverse Effect of Coronaviruses and Multiple Organ Damage**: A few days after of its entrance into the host tissue, SARS-CoV-2 virus replicates and initiates infection leading to mild symptoms following failure of innate immunity to combat it (56). The coronary respiratory syndrome induces dysregulated immune response causing tissue damage in some patients. Recovery from the virus is expected if adaptive immune system succeeds in decreasing the viral load (57). A study on 109 deceased COVID-19 patients showed multiple organ damage including heart in all patients (53).

In addition to lung damage, COVID-19 infection is known to affect multiple other organs such as heart, blood vessels, kidney, intestine, liver, brain, and the reproductive system that can manifest as a variety of clinical symptoms (48). This multiple organ damage is mediated by lung epithelial cell ACE2 receptors and multiple organ inflammation induced by pro-inflammatory cytokines released during cytokine storms and crosstalk among organs (Figure 5) (58).

**Adverse Effects of COVID-19 on the Heart**: Among the seven known strains of human Coronaviruses that affect respiratory tract, SARS-CoV-2 has also been associated with heart damage (59). Surprisingly, the virus has been reported to cause heart damage that leads to death without any obvious signs of respiratory distress and even in people without any prior cardiovascular disease (CVDs) history (60). Acute or protracted heart failure, myocarditis, cardiac cell necrosis and arrhythmias are some of the complications that have been observed in the cardiac system following COVID-19 infections (59). People with mild or no COVID-19 infection symptoms have also been reported to exhibit such complications (59). However, a high risk of mortality due to COVID-19 infections was observed in patients with CVDs (52,60).The data obtained from Wuhan, Italy and the United States of America reported that CVD is the most common cause of death of COVID-19 patients (52,61).

COVID-19 is reported to induce direct insults to myocardial cells and their function which is demonstrated by elevation of CVD biomarker level such as plasma troponin, C-reactive protein and N-terminal pro-brain natriuretic peptide (17). About 36% of Covid-19 patients showed an elevated level of troponin I as found in a study conducted on 2736 patients admitted to the Mount Sinai Health System in New York (61). In another study of 101 Covid-19 patients in Sichuan, China, 15.8% of the patients showed a plasma troponin I level greater than the normal (52,62). This increases in the level of plasma troponin in Covid-19 patients can indicate cardiovascular complication of COVID-19.

Besides the uncertainty of the specific mechanisms involved in myocardial injury induced by SARS-CoV-2 infection, a study speculated that SARS- CoV-2 can cause heart damage in two ways (63). The first way could be the direct viral invasion of the myocardial tissue that progresses to heart damage through ACE2 receptors (64). The second is through evoking cytokine storm which causes cardiac injuries (52).

**Biological Molecules involved in Heart Complications in COVID-19 patients**: The highly expressed ACE2 in the heart makes it the second major organ highly affected by SARS-CoV-2 after the lungs (65). The SARS-CoV-2 invasion of myocardial cells is facilitated via ACE2 receptors in pericytes and cause elevated macrophage infiltration, capillary endothelial cell dysfunction, and decreased ACE2 expression (66). Cardiac dysfunction and arrhythmias are also the consequences of myocardial injury in COVID-19 patients (67).

Following infection of patients with COVID-19, inflammatory cytokines are persistently elevated and cause a decrease of coronary blood flow and oxygen supply, micro thrombogenesis, and degradation of the coronary plaque which paves way for myocardial injury and heart failure (68). This hyper inflammation of myocardial cells resulting from the cytokines storm are known to exacerbate the inflammatory responses (17). An elevated level of cardiovascular markers such as kinase, creatinine, cardiac troponin I, brain natriuretic peptide, lactate dehydrogenase and other markers like, aspartate aminotransferase, and D-dimer concentration were observed in COVID-19 patients following inflammation of cardiac cells (69). The increase in the level of these molecules suggest the injury of cardiac muscle following the complications that can occur during the course of the disease.

**ACE2 in Cardiac Muscle**: The extent of viral spike protein interaction with host cells via ACE2 receptors determines the severity of COVID -19 (65). ACE2 receptors are highly expressed in the lungs and heart making these two organs the primary targets of the virus. The severity of cardiac muscle damage and progression of the diseases to respiratory distress and heart failure depends on the viral load in the muscle (70). This occurs when SARS-CoV-2 virus targets ACE2 which may in turn elevate the level of angiotensin II via nicotinamide adenine dinucleotide phosphate (NADPH) oxidase activity (17). This causes chronic myocardial hypoxia and leads to endothelial dysfunction.

**Nitric Oxide (NO)**: The damaging effect of COVID-19 on the cardiovascular system could also be associated with nitric oxide availability and its level in the myocardial cell. The bioavailability of nitric oxide is related to the level of ACE2 whereby its deficiency can result in decreased nitric oxide production and an endothelial nitric oxide synthase (eNOS) expression which can regulate blood pressure and vascular injury in cardiovascular system (71). Moreover, ACE2 deficiency in the COVID-19 patients can result in elevated levels of angiotensin II which in turn increases the production of cyclic guanosine 3′,5′-monophosphate (cGMP), degrading phosphodiesterases. This results in reduced level of cGMP in the cardiac cell induces myocardial injury and causes vascular dysfunction (72). However, there is uncertainty about the percentage contribution of SARS-CoV2-induced myocardial injury via direct viral invasion or indirect systemic toxicity (17).

**Adverse Effects of Coronaviruses on the Liver**: Because of its anatomical location and immense metabolic activity, the liver is frequently exposed to dietary antigens, toxicants, various viruses and other pathogens. A recent study showed that liver is exposed to varying degrees of damage in patients with COVID-19 (45). Studies have shown that the liver is also potentially susceptible to adverse effects of COVID-19 infection (73). Such adverse events manifest as alteration in the plasma concentration of liver function biomarkers such as alanine aminotransferase, aspartate aminotransferase and bilirubin in COVID-19 patients (64,74).

There is uncertainty on the mechanisms involved in hepatocellular damage in COVID-19 patients. It is unclear whether the SARS-COV-2 virus invasion of liver cells or immune response to COVID-19 infections or the prescribed medications cause the hepatic complications (74). However, some studies suggested that the viral leakage into hepatocytes, bile duct cells and presence of ACE2 receptors on these cells may predispose the liver to become susceptible to SARS- CoV-2 infection and eventually cause hepatic injury (74). COVID-19 induced liver impairment and dysfunction is supported by the presence of ACE2 on the hepatic cells that facilitates the binding and entry of SARS- CoV-2 into the liver cells (42).

According to a report by Aguila et al. (2020), the identification of SARS-CoV-2 RNA in the stool of COVID-19 patients with diarrhea implies the possibility of liver exposure to the virus (75). Besides, COVID-19 induced immune response inflammation and pneumonia-associated hypoxia may be implicated in liver damage in COVID-19 patients (76). In another study on 417 COVID-19 patients, 76.3% of the patients had abnormal liver test results while 21.5% had a liver injury during their hospitalization (77). Likewise, in another study, the gamma-glutamyl transferase (GGT) and alkaline phosphatase (ALP) level were elevated in 54% and 1-8% respectively in COVID 19 hospitalized patients (64).

**Biological Molecules Involved in Hepatic Adverse Effects in COVID-19 Patients**: SARS-CoV-2 virus infection can initiate cellular immune responses which cause further immune system interference. This immune system interference and SARS-CoV-2 virus induced effects cause collateral damage of hepatic cells which results in liver impairment (78). Sometimes, liver damage may occur even in the absence of appreciable SARS-CoV-2 viral load just perhaps due to the inflammatory response cells by cytotoxic T cells and Kupffer cells (79). The involvement of COVID-19 in liver disease complication is evidenced by elevated production of non-specific inflammatory markers such as virus-induced cytokine storm, neutrophil to lymphocyte counts, activation of coagulation and fibrinolysis pathway and high levels of ferritin in COVID-19 patients hepatic system (76). Moreover, the elevation in the level of hepatic enzymes such as AST and ALT and activation of circulatory cytokines exhibited in COVID-19 patients may also imply viral infection of the hepatic system (80).

However, there is speculation that hepatotoxicity or liver injury induced in COVID-19 patients may be due to prescribed medicines such as antibiotics and antiretroviral drugs (79). Liver injury in COVID-19 patients may be caused by direct liver or bile cells invasion by SARS-COV-2 virus via ACE2 receptor which may later be aggravated by hyper inflammation, cytokine storm and drug-induced damage (5,6,17,53). Therefore, COVID-19 patients treatment with hepatoprotective medicines is recommended to tackle the progression of the virus from damaging the liver (17).

**Conclusion**: In conclusion, COVID-19 impacted the global economy, health and life styles. The infection caused not only severe lung injury and damage but also multiple organ complications and dysfunction. Heart and hepatic tissue are among these organs affected by adverse effect of SARS-CoV-2 infection. The downregulation of membrane-bound active ACE2 induced by SARS-CoV-2 infection facilitates the virus to easily invade the host. The decrease in ACE2 can reduce the level of nitric oxide production and an endothelial nitric oxide synthase (eNOS) expression in the cardiac muscle. The decrease of the level of nitric oxide can affect regulation of blood pressure and vascular injury and causes cardiovascular system diseases. Inflammatory response cells by cytotoxic T cells and Kupffer cells, cytokine storm and direct liver or bile cells invasion by SARS-COV-2 virus via ACE2 receptor are the major mechanisms by which SARS-COV-2 virus affect the hepatic system. The severity of the infection is highest in individuals with deficient ACE2 expression. Individuals with certain pre-existing health complications are more susceptible to severe COVID-19 infection. A combination of collaborative direct and indirect pathogenic factors may also contribute to the severity of COVID-19 infection and pathogenesis. Therefore, saturated national healthcare systems that maintain preventive measures remain crucial to limit the rapid spread of the virus and potential COVID-19-induced organ injuries.

**Future perspectives**: Since genetic mutations are the major factor affecting the life-cycle pathways of SARS-CoV-2 which make it more infectious and lethal to humans, continual monitoring of the virus genetic modifications is very important. A more detailed study focused on the mechanism of the virus invasion and pathogenesis must be conducted to develop appropriate preventive medicine. Furthermore, a personalized therapeutic system should be designed that will help prevent the viral cell entry and replication and boost immunomodulatory and tissue reparative capacity of the patient. Therefore, multiple target therapeutic protocols are intended to impair the mechanisms of SARS-CoV-2 virus multiple organ invasion and infection. Advanced molecular based treatment strategies should also be designed to significantly reduce or prevent multiple organ injury, dysfunction and related deaths.

## Figures and Tables

**Figure 6:**
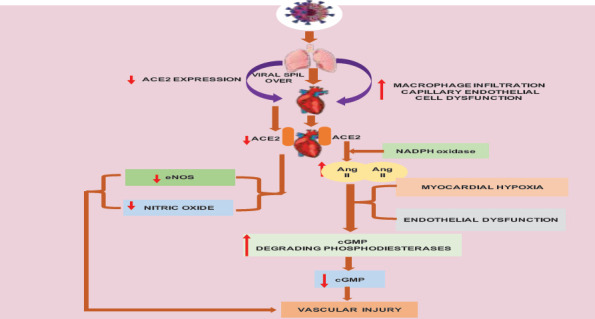
Summary of mechanisms of COVID-19 progression to the Heart: Upward red arrow: Upregulation of gene expression ; downward red arrow: down regulation of the gene expression. After COVID-19 enters into lung epithelial cells, it will undergo replication and viral progeny will be released. The spillover of these viral progeny can suit the condition for the entrance of the virus into cardiac systems. The available ACE2 heart muscle facilitate the entrance of the virus into the heart muscle and the virus directly interfere with the heart and its physiology. In other way, the expression of ACE2 downregulate on the lung epithelial cell and the increase in the microphage infiltration can also contribute for SARS-COV-2 infection to lead to the heart. The decrease in ACE2 expression on lung epithelial cells decreases ACE2 level in cardiac muscle which in turn cause the decrease in the level of eNOS and expression Nitric Oxide in cardiac system. This results in heart vascular injury. In contrary, the decreased availability of ACE2 on the heart muscle stimulate angiotensin II enzyme through NADPH oxidase which cause Myocardial Hypoxia and endothelial dysfunction. Similarly, AngII enzyme stimulation in turn stimulates cGMP degrading phosphodiesterases that reduce the availability of cGMP and finally results in vascular injury and dysfunction

## References

[1] Christian MD, Poutanen SM, Loutfy MR, Muller MP, Low DE (2004). Severe acute respiratory syndrome. Clin Infect Dis.

[2] Fehr AR, Channappanavar R, Perlman S (2017). Middle East respiratory syndrome: emergence of a pathogenic human coronavirus. Annual Review of Medicine, 68, 387–399. coronavirus. Annu Rev Med.

[3] Al-Qahtani AA (2020). Severe Acute Respiratory Syndrome Coronavirus 2 (SARS-CoV-2): Emergence, history, basic and clinical aspects. Saudi J Biol Sci.

[4] Harapan H, Itoh N, Yufika A, Winardi W, Keam S, Te H (2020). Coronavirus disease 2019 (COVID-19): A literature review. J Infect Public Health.

[5] Dhama K, Khan S, Tiwari R, Sircar S, Bhat S, Malik YS (2020). Coronavirus disease 2019-COVID-19. Clin Microbiol Rev.

[6] Naqvi AAT, Fatima K, Mohammad T, Fatima U, Singh IK, Singh A (2020). Insights into SARS-CoV-2 genome, structure, evolution, pathogenesis and therapies: Structural genomics approach. Biochim Biophys Acta (BBA)-Molecular Basis Dis.

[7] Shereen MA, Khan S, Kazmi A, Bashir N, Siddique R (2020). COVID-19 infection: Emergence, transmission, and characteristics of human coronaviruses. J Adv Res.

[8] Zhan XY, Zhang Y, Zhou X, Huang K, Qian Y, Leng Y (2020). Molecular Evolution of SARS-CoV-2 Structural Genes: Evidence of Positive Selection in Spike Glycoprotein. bioRxiv.

[9] Brian DA, Baric RS, Enjuanes L (2005). Coronavirus Genome Structure and Replication BT -Coronavirus Replication and Reverse Genetics.

[10] Giri B, Pandey S, Shrestha R, Pokharel K, Ligler FS, Neupane BB (2021). Review of analytical performance of COVID-19 detection methods. Anal Bioanal Chem.

[11] Singh D, Yi S V (2021). On the origin and evolution of SARS-CoV-2. Exp Mol Med.

[12] Zhao J, Cui W, Tian B ping (2020). The potential intermediate hosts for SARS-CoV-2. Front Microbiol.

[13] Wang HY, Li XL, Yan ZR, Sun XP, Han J, Zhang BW (2020). Potential neurological symptoms of COVID-19. Ther Adv Neurol Disord.

[14] Yanez ND, Weiss NS, Romand JA, Treggiari MM (2020). COVID-19 mortality risk for older men and women. BMC Public Health.

[15] Lakhani JD, Kapadia S, Choradiya R, Gill RP, Lakhani SJ (2021). COVID-19 and Multiorgan Dysfunction Syndrome. Fight COVID-19 Pandemic.

[16] Sourij H, Aziz F, Bräuer A, Ciardi C, Clodi M, Fasching P (2021). COVID-19 fatality prediction in people with diabetes and prediabetes using a simple score upon hospital admission. Diabetes, Obes Metab.

[17] Kuppusamy M, Wankhar W, Gurugubelli KR, Mahadevappa VH, Lepcha L, kumar Choudhary A (2021). Angiotensin-converting enzyme 2 (ACE2): COVID 19 gate way to multiple organ failure syndromes. Respir Physiol Neurobiol.

[18] Organization WH (2020). Naming the coronavirus disease (COVID-19) and the virus that causes it. Brazilian J Implantol Heal Sci.

[19] Zhu H, Wei L, Niu P (2020). The novel coronavirus outbreak in Wuhan, China. Glob Heal Res Policy.

[20] Gorbalenya AE, Baker SC, Baric RS, de Groot RJ, Drosten C, Gulyaeva AA (2020). The species Severe acute respiratory syndrome-related coronavirus: classifying 2019-nCoV and naming it SARS-CoV-2. Nat Microbiol.

[21] Özdemir Ö (2020). Coronavirus disease 2019 (COVID-19): diagnosis and management. Erciyes Med Journal/Erciyes Tip Derg.

[22] Payne S (2017). Family coronaviridae. Viruses.

[23] Gupta A, Madhavan M V, Sehgal K, Nair N, Mahajan S, Sehrawat TS (2020). Extrapulmonary manifestations of COVID-19. Nat Med.

[24] Cheng VCC, Lau SKP, Woo PCY, Yuen KY (2007). Severe acute respiratory syndrome coronavirus as an agent of emerging and reemerging infection. Clin Microbiol Rev.

[25] Azer SA (2020). COVID-19: pathophysiology, diagnosis, complications and investigational therapeutics. New Microbes New Infect.

[26] Worobey M, Levy JI, Serrano LM, Crits-Christoph A, Pekar JE, Goldstein SA (2022). The Huanan Seafood Wholesale Market in Wuhan was the early epicenter of the COVID-19 pandemic. Science (80-).

[27] Taskin Tok T, Tatar G, Tugba TT (2017). Structures and functions of coronavirus proteins: molecular modeling of viral nucleoprotein-international journal of virology & infectious diseases international journal of virology & infectious diseases. Int J Virol Infect Dis.

[28] Aleem A, Samad ABA, Slenker AK (2022). Emerging variants of SARS-CoV-2 and novel therapeutics against coronavirus (COVID-19). StatPearls [Internet].

[29] Auwaerter MDP (2022). Coronavirus COVID-19 (SARS-CoV-2) [Internet].

[30] UKHSA (2021). SARS-CoV-2 variants of concern and variants under investigation in England- Technical briefing 31. Sage.

[31] Mlcochova P, Kemp SA, Dhar MS, Papa G, Meng B, Ferreira IATM (2021). SARS-CoV-2 B. 1.617. 2 Delta variant replication and immune evasion. Nature.

[32] Jassat W, Karim SSA, Mudara C, Welch R, Ozougwu L, Groome MJ (2022). Clinical severity of COVID-19 in patients admitted to hospital during the omicron wave in South Africa: a retrospective observational study. Lancet Glob Heal.

[33] Pulliam JRC, van Schalkwyk C, Govender N, von Gottberg A, Cohen C, Groome MJ (2022). Increased risk of SARS-CoV-2 reinfection associated with emergence of Omicron in South Africa. Science (80-).

[34] Lau SKP, Li KSM, Tsang AKL, Shek CT, Wang M, Choi GKY (2012). Recent transmission of a novel alphacoronavirus, bat coronavirus HKU10, from Leschenault's rousettes to pomona leaf-nosed bats: first evidence of interspecies transmission of coronavirus between bats of different suborders. J Virol.

[35] Fehr AR, Perlman S (2015). Coronaviruses: an overview of their replication and pathogenesis. Coronaviruses.

[36] Wu A, Peng Y, Huang B, Ding X, Wang X, Niu P (2020). Genome composition and divergence of the novel coronavirus (2019-nCoV) originating in China. Cell Host Microbe.

[37] Lu R, Zhao X, Li J, Niu P, Yang B, Wu H (2020). Genomic characterisation and epidemiology of 2019 novel coronavirus: implications for virus origins and receptor binding. Lancet.

[38] Chan JFW, Kok KH, Zhu Z, Chu H, To KKW, Yuan S (2020). Genomic characterization of the 2019 novel human-pathogenic coronavirus isolated from a patient with atypical pneumonia after visiting Wuhan. Emerg Microbes Infect.

[39] Kumar S, Nyodu R, Maurya VK, Saxena SK (2019). Morphology, Genome Organization, Replication, and Pathogenesis of Severe Acute Respiratory Syndrome. Coronavirus 2.

[40] Trougakos IP, Stamatelopoulos K, Terpos E, Tsitsilonis OE, Aivalioti E, Paraskevis D (2021). Insights to SARS-CoV-2 life cycle, pathophysiology, and rationalized treatments that target COVID-19 clinical complications. J Biomed Sci.

[41] Zhang Q, Xiang R, Huo S, Zhou Y, Jiang S, Wang Q (2021). Molecular mechanism of interaction between SARS-CoV-2 and host cells and interventional therapy. Signal Transduct Target Ther.

[42] Hoffmann M, Kleine-Weber H, Schroeder S, Krüger N, Herrler T, Erichsen S (2020). SARS-CoV-2 Cell Entry Depends on ACE2 and TMPRSS2 and Is Blocked by a Clinically Proven Protease Inhibitor. Cell.

[43] Lan J, Ge J, Yu J, Shan S, Zhou H, Fan S (2020). Structure of the SARS-CoV-2 spike receptor-binding domain bound to the ACE2 receptor. Nature.

[44] Bosch BJ, Smits SL, Haagmans BL (2014). Membrane ectopeptidases targeted by human coronaviruses. Curr Opin Virol.

[45] Hikmet F, Méar L, Edvinsson À, Micke P, Uhlén M, Lindskog C (2020). The protein expression profile of ACE2 in human tissues. Mol Syst Biol.

[46] Murgolo N, Therien AG, Howell B, Klein D, Koeplinger K, Lieberman LA (2021). SARS-CoV-2 tropism, entry, replication, and propagation: Considerations for drug discovery and development. PLOS Pathog.

[47] Pizzato M, Baraldi C, Boscato Sopetto G, Finozzi D, Gentile C, Gentile MD (2022). SARS-CoV-2 and the host cell: A tale of interactions. Front Virol.

[48] Lopes-Pacheco M, Silva PL, Cruz FF, Battaglini D, Robba C, Pelosi P (2021). Pathogenesis of multiple organ injury in COVID-19 and potential therapeutic strategies. Front Physiol.

[49] Jackson CB, Farzan M, Chen B, Choe H (2022). Mechanisms of SARS-CoV-2 entry into cells. Nat Rev Mol Cell Biol.

[50] Wang K, Chen W, Zhou YS, Lian JQ, Zhang Z, Du P (2020). SARS-CoV-2 invades host cells via a novel route: CD147-spike protein. biorxiv.

[51] Gadanec LK, McSweeney KR, Qaradakhi T, Ali B, Zulli A, Apostolopoulos V (2021). Can SARS-CoV-2 virus use multiple receptors to enter host cells?. Int J Mol Sci.

[52] Huang C, Wang Y, Li X, Ren L, Zhao J, Hu Y (2020). Clinical features of patients infected with 2019 novel coronavirus in Wuhan, China. Lancet.

[53] Kordzadeh-Kermani E, Khalili H, Karimzadeh I (2020). Pathogenesis, clinical manifestations and complications of coronavirus disease 2019 (COVID-19). Future Microbiol.

[54] Perlman S, Netland J (2009). Coronaviruses post-SARS: update on replication and pathogenesis. Nat Rev Microbiol.

[55] Hasanvand A (2022). COVID-19 and the role of cytokines in this disease. Inflammopharmacology.

[56] Cao W, Li T (2020). COVID-19: towards understanding of pathogenesis. Cell Res.

[57] Diamond MS, Kanneganti TD (2022). Innate immunity: the first line of defense against SARS-CoV-2. Nat Immunol.

[58] Makaremi S, Asgarzadeh A, Kianfar H, Mohammadnia A, Asghariazar V, Safarzadeh E (2022). The role of IL-1 family of cytokines and receptors in pathogenesis of COVID-19. Inflamm Res.

[59] Topol EJ (2020). COVID-19 can affect the heart. Science (80-).

[60] Peiris S, Mesa H, Aysola A, Manivel J, Toledo J, Borges-Sa M (2021). Pathological findings in organs and tissues of patients with COVID-19: A systematic review. PLoS One.

[61] Lala A, Johnson KW, Januzzi JL, Russak AJ, Paranjpe I, Richter F (2020). Prevalence and impact of myocardial injury in patients hospitalized with COVID-19 infection. J Am Coll Cardiol.

[62] Wei W, Jiang H, Chen W, Zhou Y, Guo S, Zhong G (2020). How should we implement radiotherapy for cancer patients in China during the endemic period of COVID-19?. Radiother Oncol.

[63] Basu-Ray I, Adeboye A, Soos MP (2022). Cardiac manifestations of coronavirus (COVID-19). StatPearls [Internet].

[64] Zhang J jin, Dong X, Cao Y yuan, Yuan Y dong, Yang Y bin, Yan Y qin (2020). Clinical characteristics of 140 patients infected with SARS-CoV-2 in Wuhan, China. Allergy.

[65] Zou X, Chen K, Zou J, Han P, Hao J, Han Z (2020). Single-cell RNA-seq data analysis on the receptor ACE2 expression reveals the potential risk of different human organs vulnerable to 2019-nCoV infection. Front Med.

[66] Guo L, Yu K, Li D, Yang H, Liu L, Fan J (2020). Potential pathogenesis of multiple organ injury in COVID-19.

[67] Long B, Brady WJ, Koyfman A, Gottlieb M (2020). Cardiovascular complications in COVID-19. Am J Emerg Med.

[68] Gedefaw L, Ullah S, Leung PHM, Cai Y, Yip SP, Huang CL (2021). Inflammasome activation-induced hypercoagulopathy: impact on cardiovascular dysfunction triggered in COVID-19 patients. Cells.

[69] Cheng MP, Papenburg J, Desjardins M (2020). Original: diagnostic testing for severe acute respiratory syndrome-related. N Engl J Med.

[70] Lauer SA, Grantz KH, Bi Q, Jones FK, Zheng Q, Meredith HR (2020). The incubation period of coronavirus disease 2019 (COVID-19) from publicly reported confirmed cases: estimation and application. Ann Intern Med.

[71] Rabelo LA, Todiras M, Nunes-Souza V, Qadri F, Szijártó IA, Gollasch M (2016). Genetic deletion of ACE2 induces vascular dysfunction in C57BL/6 mice: role of nitric oxide imbalance and oxidative stress. PLoS One.

[72] Naz A, Billah M (2021). COVID-19 and coronary heart disease. Encyclopedia.

[73] Wu J, Song S, Cao HC, Li LJ (2020). Liver diseases in COVID-19: Etiology, treatment and prognosis. World J Gastroenterol.

[74] Saviano A, Wrensch F, Ghany MG, Baumert TF (2021). Liver disease and coronavirus disease 2019: from pathogenesis to clinical care. Hepatology.

[75] Aguila EJT, Cua IHY, Fontanilla JAC, Yabut VLM, Causing MFP (2020). Gastrointestinal manifestations of COVID-19: impact on nutrition practices. Nutr Clin Pract.

[76] Somers VK, Kara T, Xie J (2020). Progressive hypoxia: a pivotal pathophysiologic mechanism of COVID-19 pneumonia. Mayo Clinic Proceedings.

[77] Cai Q, Huang D, Yu H, Zhu Z, Xia Z, Su Y (2020). COVID-19: Abnormal liver function tests. J Hepatol.

[78] Bangash MN, Patel J, Parekh D (2020). COVID-19 and the liver: little cause for concern. lancet Gastroenterol Hepatol.

[79] Li D, Ding X, Xie M, Tian D, Xia L (2021). COVID-19-associated liver injury: from bedside to bench. J Gastroenterol.

[80] Boettler T, Newsome PN, Mondelli MU, Maticic M, Cordero E, Cornberg M (2020). Care of patients with liver disease during the COVID-19 pandemic: EASL-ESCMID position paper. JHEP reports.

